# Charge injection and transport properties of an organic light-emitting diode

**DOI:** 10.3762/bjnano.7.5

**Published:** 2016-01-14

**Authors:** Peter Juhasz, Juraj Nevrela, Michal Micjan, Miroslav Novota, Jan Uhrik, Lubica Stuchlikova, Jan Jakabovic, Ladislav Harmatha, Martin Weis

**Affiliations:** 1 Slovak University of Technology, Ilkovicova 3, Bratislava 81219, Slovakia

**Keywords:** activation energy, impedance spectroscopy, organic light-emitting device

## Abstract

The charge behavior of organic light emitting diode (OLED) is investigated by steady-state current–voltage technique and impedance spectroscopy at various temperatures to obtain activation energies of charge injection and transport processes. Good agreement of activation energies obtained by steady-state and frequency-domain was used to analyze their contributions to the charge injection and transport. We concluded that charge is injected into the OLED device mostly through the interfacial states at low voltage region, whereas the thermionic injection dominates in the high voltage region. This comparison of experimental techniques demonstrates their capabilities of identification of major bottleneck of charge injection and transport.

## Introduction

Since the discovery of organic electroluminescent (EL) materials such as tris(8-hydroxyquinolinato)aluminum(III) (Alq3), organic light-emitting devices (OLEDs) have drawn huge attention in electronics [1]. OLED devices are envisioned as future light sources because of possible flexibility, transparency, and low-cost large-area production; however, OLEDs have recent reached luminous efficacy over 130 lm/W [2–3] that is double of fluorescent tube efficacy (60–70 lm/W), which is the current benchmark for novel light sources [4].

Organic semiconductors have zero doping level and very low intrinsic charge density, therefore all charges in OLED device are injected from the electrodes. As a result, the energy band diagram analysis plays a key role in the study of the charge injection/transport phenomena. Although, these important ideas are well-accepted in the development of high-performance OLED devices, deep understanding of the physical processes regarding the injection and transport of charge carriers is needed for further device performance improvement. In details, the organic–organic and metal–organic interfaces determine injection properties, whereas the conductivities of organic layers limit the charge transport properties. Charge transport in organic semiconductors has been widely studied by electrical characterization techniques such as steady-state current density–voltage characteristics [5–6], or measurement in time- or frequency-domain, such as transient currents [7] and impedance spectroscopy [8–9]. Furthermore, the measurements can be extended by the temperature dependence of electrical properties which reveal the thermally activated charge behavior [10]. It should be noted here that the electrical properties of organic devices are strongly dependent of device fabrication. Therefore, a detailed comparison of results obtained at various devices is not applicable although identical materials/structures are used. Hence, the correlation between obtained results of different characterization techniques is reliable only for the same device.

This study demonstrates charge transport properties in OLED devices formed by indium tin oxide (ITO)/*N*,*N'*-di-1-naphthyl-*N*,*N*'-diphenyl-1,1'-biphenyl-4,4'-diamine (α-NPD)/Alq3/Al system. The steady-state current–voltage characteristics recorded at various temperatures have been used to evaluate the activation energy of electric conductivity. Obtained results are compared with energy band diagram to identify major energy barriers limiting the current.

## Experimental

The study of the charge transport properties of OLED devices has been done on the organic double-layer sandwiched between two electrodes ITO/α-NPD/Alq3/Al. The devices were grown on glass slides precoated with ITO with sheet resistance lower than 10 Ω/sq. The substrates were cleaned sequentially in ultrasonic bath by isopropanol and deionized water and then treated by oxygen plasma to remove organic residues. Prior to the organic material deposition the substrates were heated up to 200 °C during 30 min in vacuum better than 10^−5^ Pa. Devices were formed by sequential thermal evaporation of hole transport material α-NPD (Sigma-Aldrich) followed by a layer deposition of electroluminescent material Alq3 (Tokyo Chemical Industry). The thicknesses of α-NPD and Alq3 were 150 and 50 nm, respectively, deposited at constant deposition rate of 0.6 nm/min. The Al electrode of 100 nm in thickness has been deposited through the shadow mask. All deposition processes have been done without braking of vacuum to avoid unintended defects. The OLED active area of 4 mm^2^ was formed by overlap of ITO and Al electrodes.

All electrical characterizations have been done under vacuum in temperature range from 200 to 325 K. The steady-state current–voltage characteristics have been recorded using an Agilent Semiconductor Parameter Analyzer 4155C in the voltage range from −0.5 to 10 V. The frequency measurements have been carried out by the MODULAB MTS system with offset from −0.5 to 8 V with the probe signal AC amplitude of 10 mV. The impedance magnitude and phase frequencies ranged from 10 Hz to 1 MHz.

## Results and Discussion

**Figure 1:**
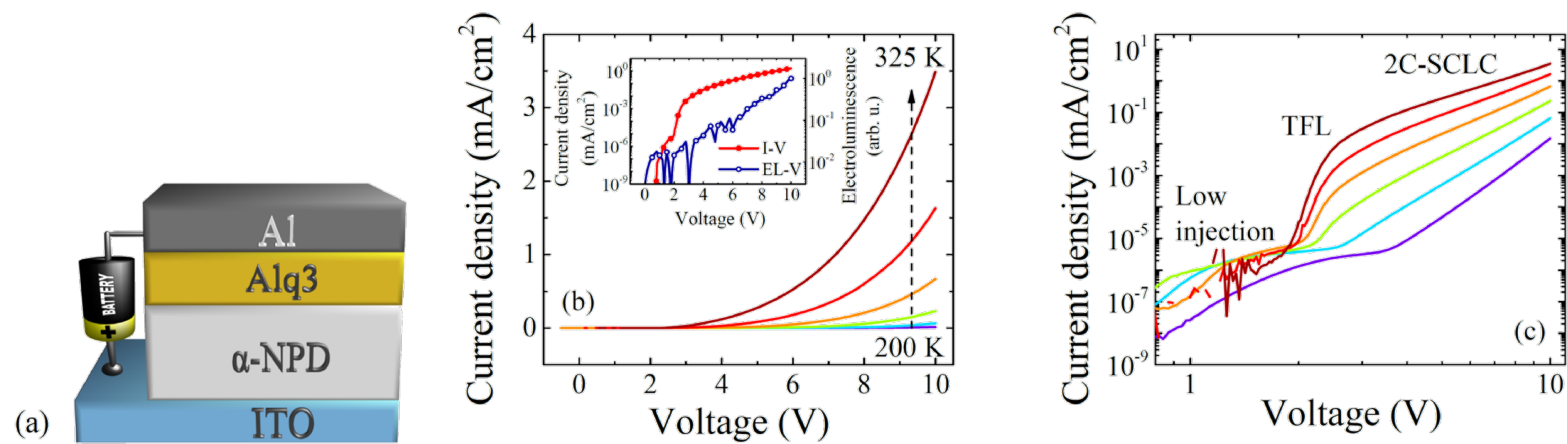
(a) Schematic diagram of the device ITO/α-NPD/Alq3/Al. Steady-state current–voltage characteristics (b) in linear scale, the inset shows current density–voltage characteristic and electroluminescence–voltage characteristic at 300 K in a semi-log scale, and (c) in log–log scale recorded over temperature range from 200 to 325 K. Abbreviations TFL and 2C-SCLC stand for trap-fill-limit and two-carrier space-charge limited conditions, respectively.

Figure 1 depicts a set of current density–voltage (*J*–*V*) characteristics recorded at various temperatures from 200 to 325 K. The OLED device exhibits rectifying property with an on/off ratio of about 10^4^ at room temperature. Note that in low-conduction materials, ohmic transport is common for the low-voltage region, whereas the space-charge limited current (SCLC) regime usually governs the carrier transport at higher voltage region. Since, the well-known Mott–Gourney square law *J* 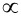 *V*^2^ is valid only for the single-carrier transport in trap-free material, in real OLED devices the power exponent of the voltage usually reaches values higher than 2. The voltage dependencies in a log–log scale can be apparently divided to three regions in accordance to the different power exponent represented by the slope of the *J*–*V* characteristics.

At voltages below 2 V the low charge injection region can be recognized, since the current density *J* follows applied voltage bias *V* linearly,

[1]
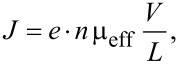


where *e*·*n* is the charge density (*e*: elementary charge, *n* charge carrier density), μ_eff_ is the effective charge mobility, and *L* the organic film thickness. Note that the effective charge mobility μ_eff_ includes charge trapping phenomenon as follows,

[2]
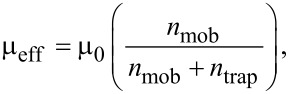


where μ_0_ is trap-free charge mobility, *n*_mob_ and *n*_trap_ are mobile and trapped charge carrier densities, respectively. In the voltage region from 2 to 3 V an abrupt increase of the current density is observed. Note that the significant rise of the current in certain voltage region only is usually assigned to the trap-filled-limit (TFL) voltage. In other words, the charge transport properties of organic films are changed due to filling of all localized states and charge carriers are no more influenced by the trapping mechanism. At voltages higher than 3 V the rise of current density slows down to *J* 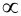 *V*^3^ and follows two-carrier space-charge limited conditions (2C-SCLC),

[3]
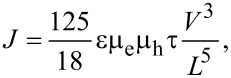


where ε is the dielectric constant of the organic film, μ_e_ and μ_h_ are electron and hole mobilities, respectively, and τ is carrier lifetime [13]. However, the 2C-SCLC model is fully valid only if no injection barrier is present at metal–organic semiconductor interface. Previous studies revealed that observed current density–voltage characteristics can be also associated with thermionic emission or Fowler–Nordheim tunneling [14–16]. Hence, to distinguish between space-charge limited current and interface limited current is required more deep analysis.

**Figure 2:**
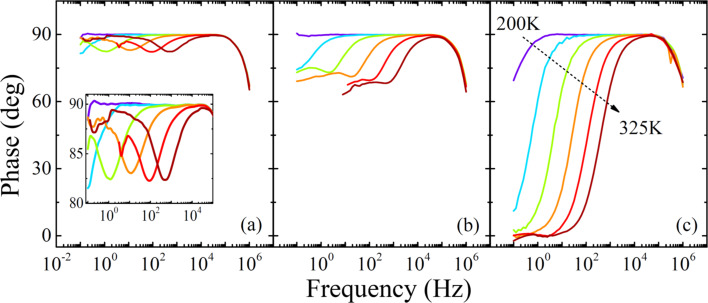
Impedance phase frequency spectra recorded in the temperature range from 200 to 325 K with the step of 25 K at voltage biases of (a) 1 V, (b) 2 V, and (c) 4 V. Inset depicts detail view of the phase spectra.

The impedance spectroscopy is well-established characterization technique used to study the dielectric layers properties in frequency domain. It can be applied to investigate the dynamics of mobile charge carriers in the bulk of the layer or at the interfacial region between two layers. Here, the impedance spectroscopy has been applied for detail characterization of different charge transport regions estimated by current density–voltage measurements, since impedance spectroscopy is capable to distinguish charge relaxation processes with dissimilar relaxation times. Figure 2 illustrates impedance phase-shift spectra in all investigated voltage regions. Note that all spectra share a common decrease of the phase in high frequency region that originates from the parasitic series resistance of about 60 Ω. Interestingly, the low-injection region is represented by the impedance spectra that exhibit two transitions between different phases, as depicted in Figure 2a. Each transition of phases stands for a different relaxation process. However, due to the low current density the phase saturates in low frequency region at 90 degree that represents capacitor-like behavior. The relaxation processes are obviously thermally stimulated, and one of the processes is significantly more sensitive to a rise of temperature. In other words, two processes have strongly dissimilar activation energies. In the voltage range that has been assigned to TFL again two relaxations are observed. However, rising the current density induces saturation of the impedance phase at the value in between capacitor-like behavior (90 degree) and resistor-like behavior (0 degree). Surprisingly, at higher voltages only one charge-relaxation process is present and the device exhibits pure resistor-like behavior in the low-frequency region.

**Figure 3:**
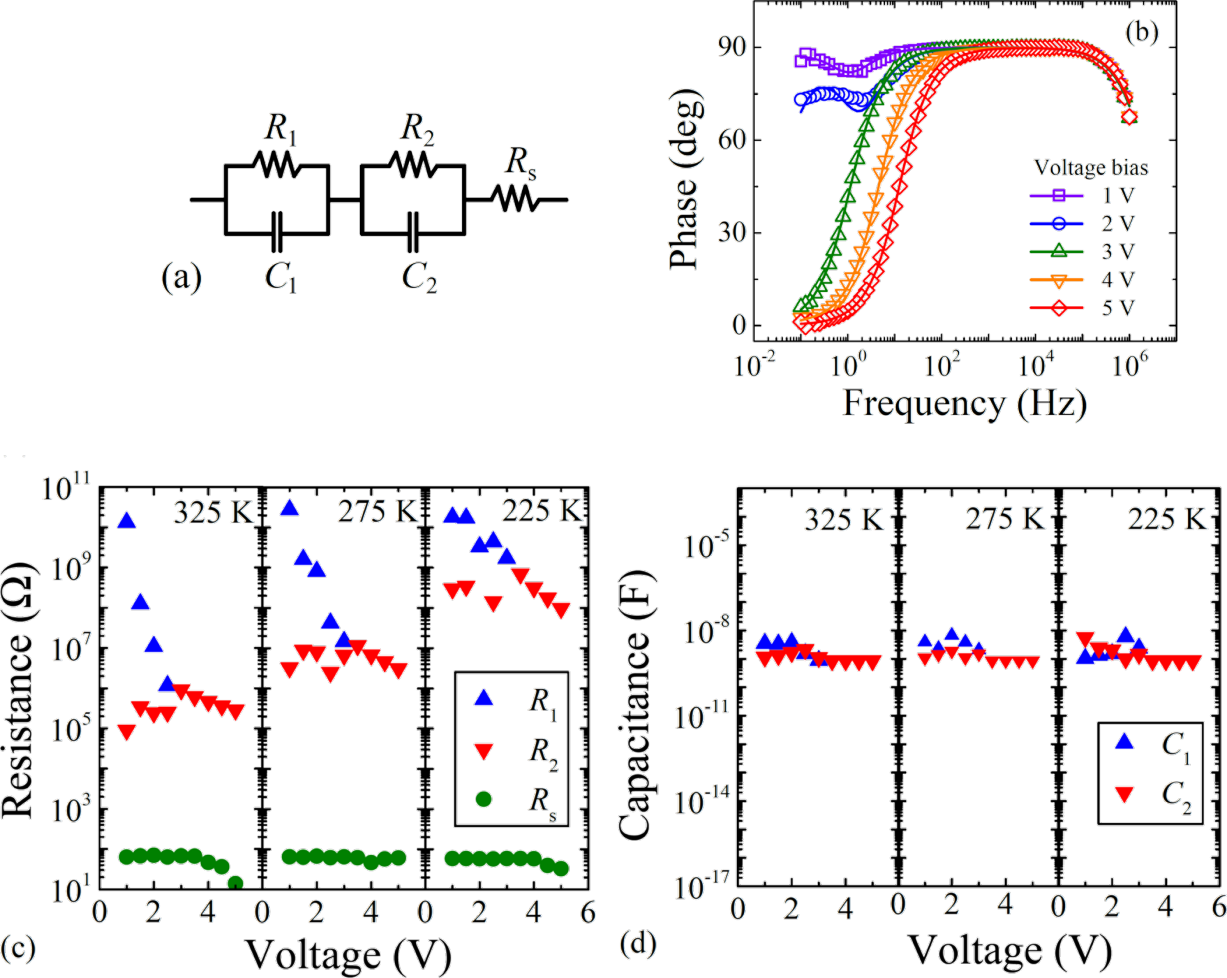
(a) Electrical equivalent circuit model used for data evaluation, (b) the comparison of recorded and fitted impedance phase spectra at temperature 250 K. Symbols represent the experiment, solid line show the equivalent circuit model evaluation. Obtained values of (c) resistances and (d) capacitances of equivalent electrical circuit.

The evaluation has been done using electrical equivalent circuits shown in Figure 3a and fitting results have been found sufficient, see Figure 3b. Obtained values of equivalent electrical circuit elements are depicted in Figure 3c and Figure 3d. The resistances *R*_1_ and *R*_2_ are related to the charge injection/transport phenomena and exhibit strong voltage dependencies. Higher resistance stands for process representing major charge injection/transport limitation in low voltage region, but it diminishes at higher voltages. On the other hand, capacitances *C*_1_ and *C*_2_ consisting of a geometric capacitance (constant contribution of approx. 1 nF) and a differential capacitance (charge-dependent contribution) are almost independent of the voltage.

Since a high current density is a characteristic feature of the 2C-SCLC region, the major charge relaxation phenomenon should be related to the charge transport. Hence, in regions of a high applied voltage the relaxation time *t*_relax_ obtained from the impedance spectra analysis has been assigned to the transit time *t*_tr_ across the organic film as *t*_tr_ 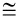 0.72*t*_relax_ [8]. The effective mobility can be evaluated in accordance to the time-of-flight model [9] as,

[4]
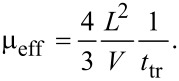


**Figure 4:**
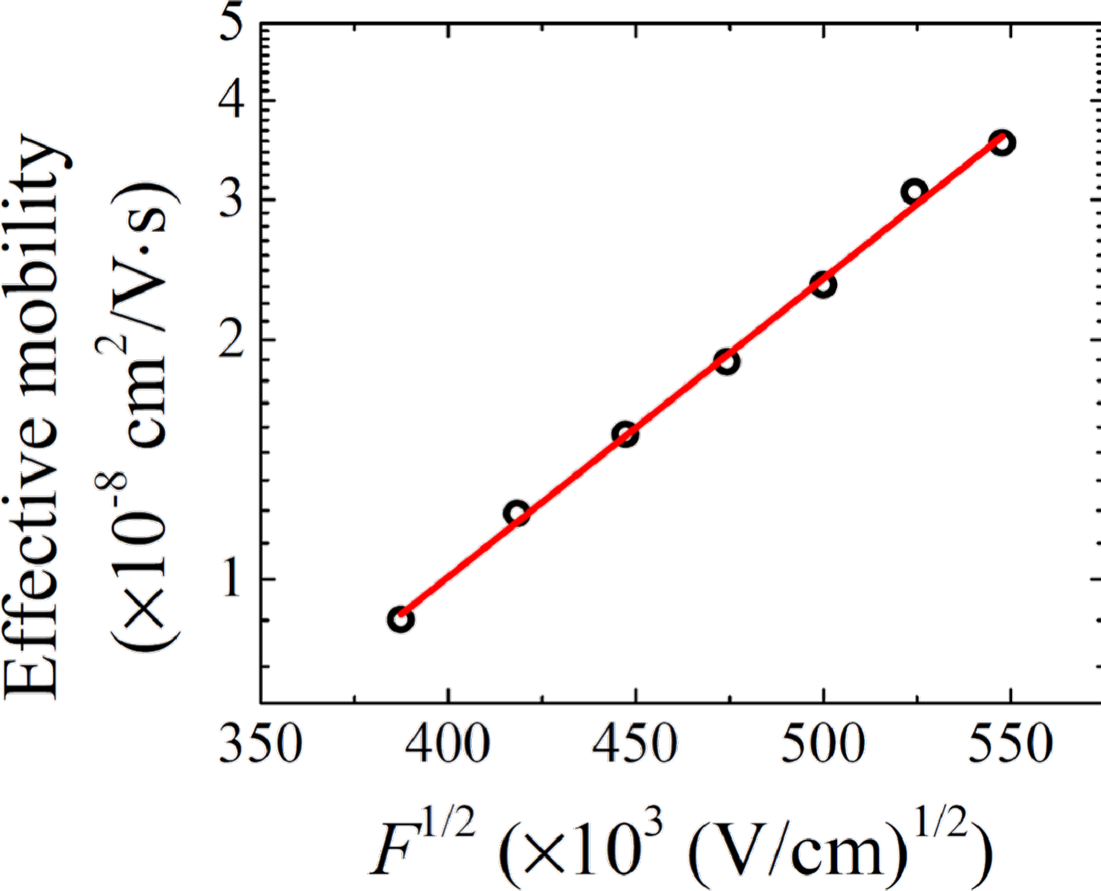
Electric field dependence of the effective mobility at the temperature of 300 K. The solid line represents the linear fit.

The effective mobility value is in good agreement with hole mobility in Alq3 [9]. Since the hole mobility of α-NPD ranges from 10^−4^ to 10^−3^ cm^2^/V·s [11–12], the hole mobility in Alq3 represents the charge transport bottleneck. It is interesting to note that estimated effective mobility follows the Poole–Frenkel dependence on the electric field,

[5]
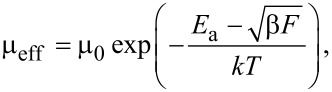


where *E*_a_ is the activation energy of the relaxation process, 
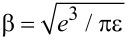
 is the Schottky parameter, *F* is the intensity of electric field, and *kT* is the thermal energy (*k*: Boltzmann constant, *T*: thermodynamic temperature), as demonstrated in Figure 4. The electric field *F* is assumed as an average field across the device *F* = *V*/*L*, which is in good agreement with zero charge contribution to the capacitance. It should be mentioned here that the Poole–Frenkel model represents the charge-carrier hopping between localized states or thermionic emission through the energy barrier (the Schottky effect), where the energy barrier *E*_a_ is suppressed by the local electric field *F* [17]. Therefore, the evaluation of the activation energies is required for further understanding the charge-relaxation processes in OLED devices.

**Figure 5:**
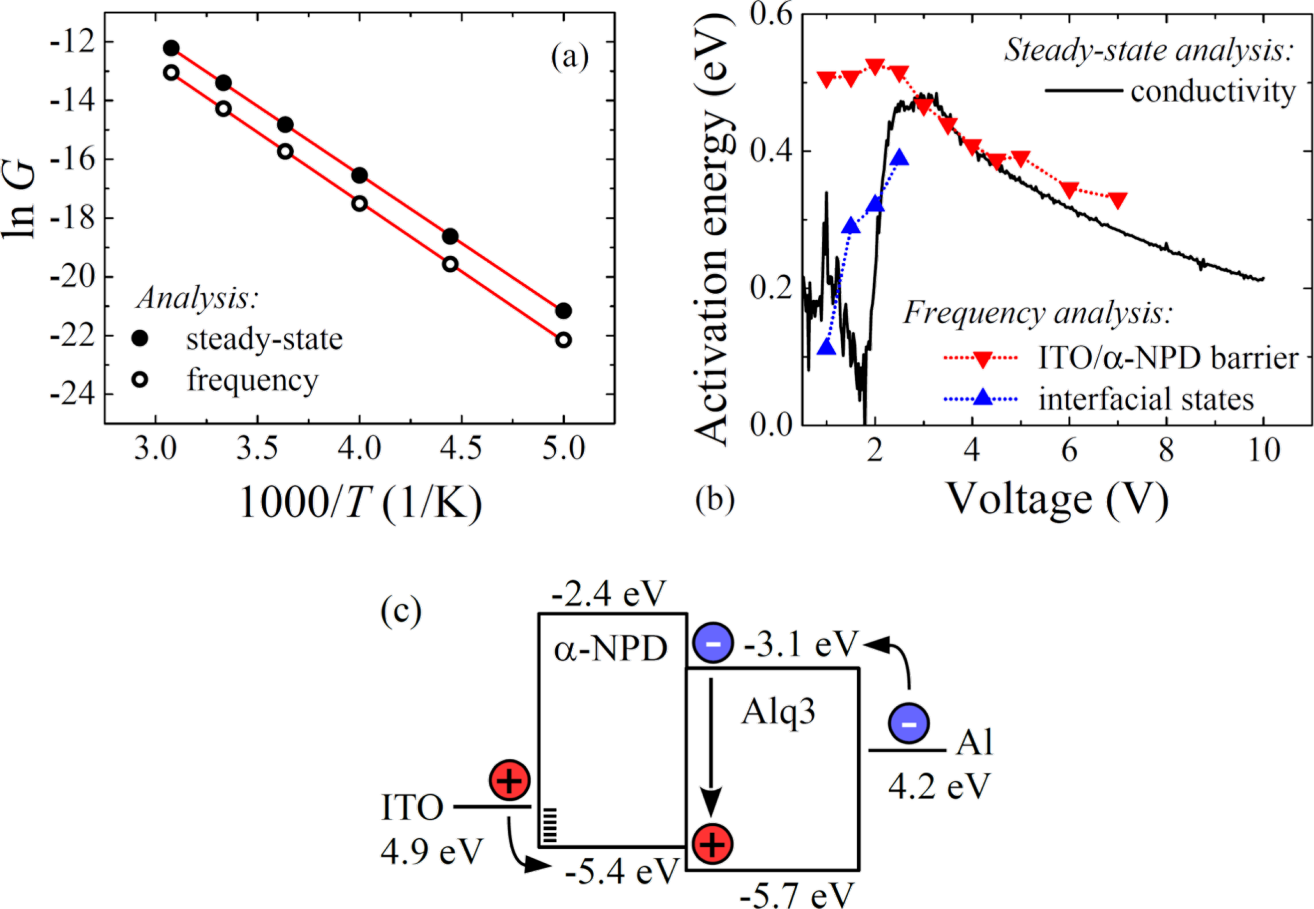
(a) Arrhenius plot of conductivities estimated from steady-state current density–voltage measurement and impedance spectroscopy spectra at voltage bias of 4 V and (b) evaluated voltage dependence of activation energies. (c) Energy band diagram model.

Charge injection/transport phenomena are thermally activated processes that follow the Boltzmann distribution. The Arrhenius plots of conductivities have been used to evaluate activation energies, as shown in Figure 5a. Since the conductivity *G* is affected by charge density and carrier mobility,

[6]
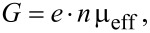


it reflects the impact of charge traps on the carrier transport as well as that of energy barriers on the carrier injection. Figure 5b depicts the voltage dependence of the activation energies estimated from the steady-state current–voltage and impedance spectroscopy conductivities. In the low-injection region two relaxations with different activation energies are observed. Note that the activation energy of about 0.5 eV is voltage-independent up to the 2C-SCLC region (3 V or higher voltage), while the other one is gradually increasing with the rise of the voltage. In the 2C-SCLC region the activations energies estimated from the steady-state and frequency analyses are in good agreement. It should be mentioned here that the decay of the activation energy in 2C-SCLC region follows the square root of the electric field as predicted by the Poole–Frenkel or thermionic emission model. As a result, this charge relaxation can be assigned to an injection of the charge over the energy barrier lowered by the external field. On the other hand, the other relaxation in the low-injection region is ascribed to the charge injection through the interfacial states. An increase of the voltage causes a gradual filling of states that represents rise of the activation energy. After the filling of all interfacial states the charge is injected only over the interfacial barrier, which stands for second charge relaxation. This assumption is supported by diminishing of interfacial state relaxation after reaching of TFL region. Since steady-state methods are sensitive only to the major bottleneck of the charge injection/transport, we can conclude that the charge is injected into the OLED device mostly through the interfacial states in the low-voltage region, whereas the thermionic injection dominates in the high-voltage region.

The energy band diagram reconstruction is required for further identification of the energy barrier origin. The work function of cleaned ITO electrodes is at a level of 4.9 eV [18–19], while the Al electrode reaches only 4.2 eV [20]. The energies of the highest occupied molecular orbitals (HOMO) and lowest unoccupied molecular orbitals (LUMO) of α-NPD and Alq3 are also known [21–22]. As a result, in accordance with the energy band diagram of the investigated OLED device, see Figure 5c, the only interface that satisfies these requirements is the ITO/α-NPD interface. Therefore, we can conclude that hole injection properties determine the performance of the ITO/α-NPD/Alq3/Al device. Furthermore, it is known that the high electron-injection barrier at the Alq3/Al interface suppresses the electron density in the OLED device and the hole density dominates in the OLED device [23]. Therefore, the holes are the main contribution to the charge injection/transport processes and activation energies of electron-related processes are not observable.

## Conclusion

This paper suggests methodology suitable for study of charge transport properties in OLED device structure ITO/α-NPD/Alq3/Al by steady-state current density-voltage measurement and impedance spectroscopy spectra. The current density-voltage characteristics revealed presence of three different regions: (i) low injection region, (ii) trap-fill-limit region, and (iii) two-carrier space-charge limited current region. The analysis of impedance spectroscopy spectra found two charge relaxations in low injection region, while the only one at higher applied voltages. The detail analysis of activation energies voltage dependence suggested that at low voltages the charge is injected by hopping through interfacial localized states, while at high voltages is dominant thermionic injection.
